# Monitoring of the sialylation and polysialylation status of sheep milk during early lactation

**DOI:** 10.3168/jdsc.2026-1050

**Published:** 2026-05-15

**Authors:** Lisa Isernhagen, Kaja Helffenstein, Anna Seidel, Bjoern Kuhla, Sebastian P. Galuska

**Affiliations:** Research Institute for Farm Animal Biology (FBN), Dummerstorf, 18196, Germany

## Abstract

The beneficial properties of sialylated biomolecules in milk are important for the optimal development of offspring. However, when assessing risk factors in food, the bioactivity of one sialic acid, *N*-glycolylneuraminic acid (Neu5Gc), is taken into account because Neu5Gc is supposed to induce proinflammatory responses and even cancer in humans. For these reasons, we determined the concentrations of the 2 main sialic acids found in mammals—Neu5Gc and *N*-acetylneuraminic acid (Neu5Ac)—in sheep milk during lactation. In addition, we examined the milk for polysialic acid (polySia), a bioactive Neu5Ac polymer. As previously observed in dairy cows, the levels of Neu5Ac, Neu5Gc and polySia decrease rapidly during the first days of lactation. However, in comparison to bovine milk, sheep milk contained significantly higher concentrations of Neu5Gc during lactation. Because of the different bioactivities of the analyzed sialic acids in mammals, the obtained results are important for human nutrition and feeding sheep offspring.

## Highlights

• Sialic acids in sheep milk rapidly decrease during early lactation.
• In contrast to other mammals, the most abundant sialic acid in sheep is Neu5Gc.
• Polysialic acid in sheep milk decreases during early lactation.
• The polySia chains in sheep colostrum are shorter than those in humans.

## Graphical Abstract

**Figure:**
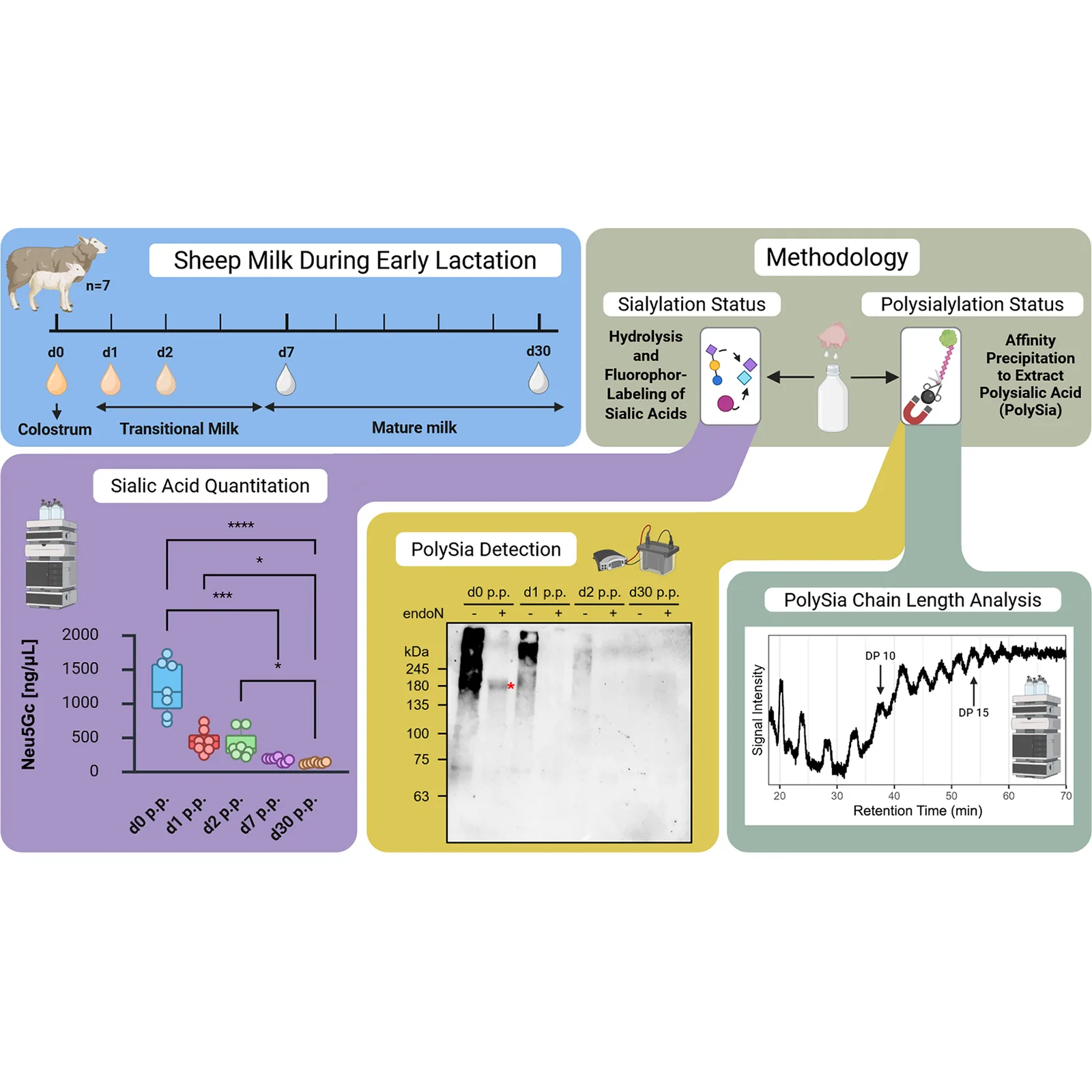
**Summary:** Sialylated biomolecules in milk are crucial for optimal offspring development. Therefore, we analyzed the sialylation and polysialylation status of sheep milk during the first month of lactation. A high-performance liquid chromatography (HPLC) approach was applied to quantify the sialic acids Neu5Ac and Neu5Gc. Although both sialic acids decreased rapidly during the initial stages of lactation, the Neu5Gc:Neu5Ac ratio remained at around 90:10. Interestingly, in large ruminants such as dairy cows, this ratio is reversed. Furthermore, we detected the bioactive carbohydrate polymer polysialic acid (polySia) in sheep milk using western blotting, which revealed a rapid decrease within the first 2 days. Chain length analysis using an HPLC-based strategy demonstrated the presence of chains longer than 17 residues, which is the critical length necessary for interaction with various bioactive molecules. These results provide valuable basic knowledge for optimizing sheep breeding and evaluating food intended for human consumption. Created with BioRender.com.

The sialic acids of glycoconjugates in the cellular glycocalyx regulate various physiological processes (Gagneux et al., 2022). They are located at the outermost position of glycans and act as binding sites for endogenous cell receptors. However, several pathogens, including influenza viruses, are also equipped with glycan-binding receptors that use sialylated glycan motifs as docking sites. As a part of the innate immune system, epithelial cells secrete highly sialylated mucins that block the binding sites of pathogen receptors and, consequently, their adhesion. During the critical early stages of life, when the offspring's immune system is not yet fully developed, sialylated milk oligosaccharides and other sialylated milk glycoconjugates support this inhibitory mechanism (Hobbs et al., 2021). Furthermore, sialic acids positively affect the microbiota as well as the development of the immune and nervous systems in the offspring (Bode, 2015). Intriguingly, polysialyltransferases can also form linear homopolymers of sialic acids using *N*-acetylneuraminic acid (**Neu5Ac**). These polysialic acid (**polySia**) chains play an important role during the development of numerous organs and in regulating the neuronal and the immune system (Colley et al., 2014; Schnaar et al., 2014). For instance, polySia can bind to and modulate the activity of lactoferrin, which is one of the most abundant bioactive proteins in milk (Kühnle et al., 2019c). Therefore, sialic acids and their polymers are interesting biomolecules to use in infant formula and functional foods (Robinson, 2019).

However, the type of sialic acid is important when considering farm animal milk for human consumption. Although humans only synthesize Neu5Ac, other mammals also produce and use *N*-glycolylneuraminic acid (**Neu5Gc**; Varki, 2010). Remarkably, ingested Neu5Gc from animal products serves as substrate for human sialyltransferases resulting in Neu5Gc-decorated glycans (Tangvoranuntakul et al., 2003). This triggers a “xeno-autoantibody” response, which is associated with inflammation and cancer (Dhar et al., 2019). Although the sialylation status of dairy cow milk has been well studied, little is known about the content and changes in sheep milk throughout lactation.

For these reasons, we aimed to quantify the concentrations of the sialic acids Neu5Gc and Neu5Ac in sheep colostrum and milk during the first month after parturition. In addition, we analyzed the colostrum, transitional, and mature milk for polySia because of the beneficial bioactivity of Neu5Ac polymers. We hypothesized that significant differences in sialic acid composition have developed during evolution between the colostrum, transitional milk, and mature milk of small and large ruminants, such as sheep and dairy cows, respectively.

Sheep (*Ovis aries*) milk samples were obtained from randomly selected East Friesian sheep (n = 7; age, 4–6 yr; 81.5 kg BW) fed hay (1,526 g of DM/d) and wheat (936 g of DM/d) at the FBN (Dummerstorf, Germany). Milk samples were collected within 12 h after parturition (colostrum, day 0 postpartum [**p.p.**]) and in the morning on d 1, 2, 7, and 30 p.p. Sampling was approved by the ethics committee of the State Department for Agriculture, Food Security and Fisheries Mecklenburg-Western Pomerania, Rostock, Germany (LALLF M-V 7221.3-1-012/23). The milk samples (10–15 mL) were frozen immediately after hand milking and stored at −20°C until use.

For sialic acid quantification, the 1,2-diamino-4,5-methylenedioxybenzene (**DMB**)-based method was applied (Hara et al., 1987) using 0.5 µL of whole milk. First, sialic acids were released in 0.2 *N* trifluoroacetic acid (**TFA**) for 4 h at 80°C and fluorescently labeled with 2.7 m*M* DMB in reaction-buffer (9 m*M* sodium hydrosulfite, 0.5 *M* β-mercaptoethanol, and 20 m*M* TFA) for 2 h at 55°C. As previously described, the hydrolysis conditions applied were optimized for quantifying mono-, oligo- and polysialylated glycoconjugates in biological samples (Inoue and Inoue, 2003). The DMB-labeled sialic acids were analyzed using a Shimadzu HPLC system (Shimadzu, Duisburg. Germany) with a C18 column (Superspher 100 RP-18 decapped LiChroCART 250–2, Sigma-Aldrich, Taufkirchen, Germany) and an RF-10A XL fluorescence detector at excitation wavelength of 372 nm and emission wavelength of 456 nm as described previously (Hinterseher et al., 2022; Isernhagen et al., 2024; Isernhagen et al., 2025). For quantification, a calibration line of commercially available standards of Neu5Ac (Carbosynth Limited, Compton, UK) and Neu5Gc (Sigma-Aldrich) was used in addition to ketodeoxynonulosonic acid (Sigma-Aldrich) as an internal standard.

Statistical analysis and data visualization was performed using the Graphs function from BioRender.com, where all statistical tests integrated are using R (https://www.r-project.org/, version 4.2.2.). Because the Shapiro–Wilk normality test performed by BioRender.com suggested a not normal distribution, the results were analyzed with Dunn's multiple comparison test, with the significance levels indicated as follows: *P < 0.05, **P < 0.01, ***P < 0.001, ****P < 0.0001.

Sialic acid analysis revealed that the Neu5Ac concentration was highest in colostrum, with a rapid decrease occurring in the days following parturition. Although 255.2 ± 111.8 ng/µL (mean ± SE) of Neu5Ac were present in colostrum, only 28.8 ± 8.7 ng/µL were detected on d2 p.p., with the lowest concentration (8.6 ± 1.0 ng/µL) observed in mature milk at the end of the first month (Figure 1A and Table 1). Unlike milk from most other mammals, which primarily contains Neu5Ac, the predominant sialic acid in East Friesian sheep milk is Neu5Gc. The highest concentration of Neu5Gc was found on d 0 p.p. at 1,237.9 ± 150.5 ng/µL, followed by a reduction to 412.7 ± 76.4 ng/µL on d 2 p.p. and 133.1 ± 5.7 ng/µL on d 30 p.p. (Figure 1B and Table 1). This demonstrates a rapid and significant decrease in sialylation in ovine milk during the first month of lactation, with high individual variability in the first few days after parturition. However, the variability between the 7 analyzed animals decreased after the transition phase.

**Figure 1:**
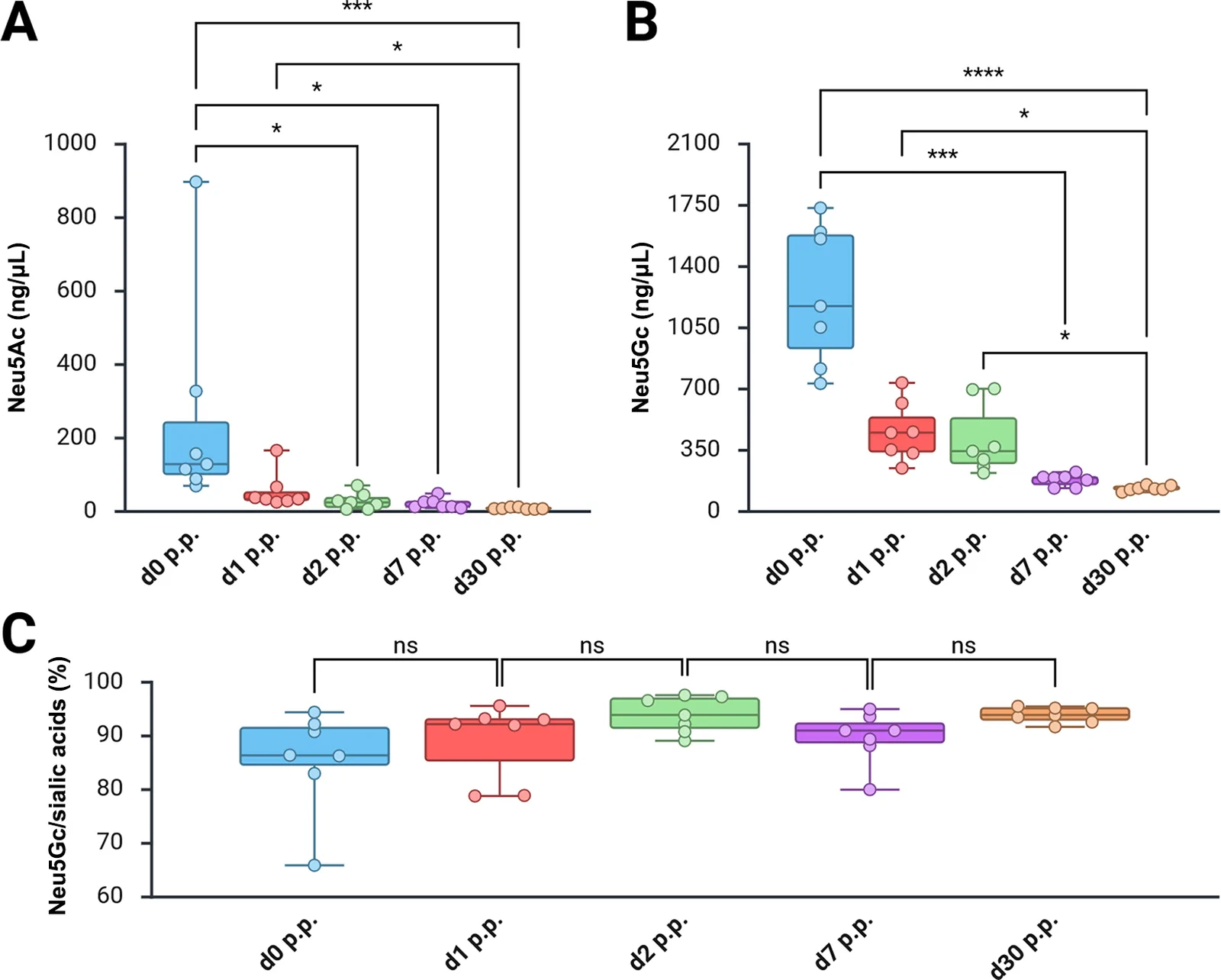
Analysis of sialic acids in sheep milk during early lactation. Box and whisker plots (median; minimum to maximum; lower whisker: minimum value to first quartile [Q1]; upper whisker: third quartile [Q3] to maximum; midlines: median; dots: data points) showing the (A) Neu5Ac and (B) Neu5Gc concentration in milk (n = 7) in addition to (C) the ratio of Neu5Gc to the total amount of sialic acids (Neu5Ac + Neu5Gc). *P < 0.05, ***P < 0.001, ****P < 0.0001.

**Table 1:** Sialic acid quantification (n = 7; average ± SE)^1^

Average (days p.p.)	Neu5Ac (ng/μL)	Neu5Gc (ng/μL)	SA (ng/μL)	Neu5Gc/SA (%)
0	255.2 ± 111.8	1,237.9 ± 150.5	1,493.1 ± 240.9	85.6 ± 3.6
1	56.3 ± 19.0	456.6 ± 64.3	512.9 ± 72.6	89.1 ± 2.7
2	28.8 ± 8.7	412.7 ± 76.4	441.5 ± 82.7	93.9 ± 1.3
7	21.6 ± 5.3	179.7 ± 13.0	201.3 ± 16.3	89.7 ± 1.8
30	8.6 ± 1.0	133.1 ± 5.7	141.7 ± 6.2	93.9 ± 0.5

1 Neu5Ac = Neu5Ac quantitated in milk; Neu5Gc = Neu5Gc quantitated in milk; SA = sum of Neu5Ac and Neu5Gc.

When the proportion of Neu5Gc was compared with that of the complete sialic acid pool (Neu5Ac + Neu5Gc), no significant change was observed in the first month after parturition. However, the variability appeared to be higher in sheep colostrum, with 85.6% ± 3.6% Neu5Gc on d 0 p.p. and 93.9% ± 0.5% on d 30 p.p. (Figure 1C and Table 1). Thus, the ratio of the 2 sialic acids in East Friesian sheep milk was approximately 90:10 (Neu5Gc:Neu5Ac). A similarly high proportion of Neu5Gc was also observed in a previous study analyzing sialic acids in mature sheep milk (Ret et al., 2025). Such a high ratio of Neu5Gc appears to be unique to sheep. The milk of other mammals, such as cows, water buffaloes, and horses, contains concentrations of Neu5Ac that are approximately 10 times higher than those of Neu5Gc (Albrecht et al., 2014; Isernhagen et al., 2025; Ret et al., 2025). Goat milk is probably most similar to sheep milk. It has been reported that there is only a slightly higher percentage in goat milk of Neu5Ac than Neu5Gc (Albrecht et al., 2014; Chen et al., 2024; Ret et al., 2025). Therefore, the proportion of Neu5Gc in the milk of goats is also significantly lower than that detected in sheep milk at all stages of lactation (Figure 1C).

To the best of our knowledge, this is the first detailed description of the sialic acid content in sheep milk during lactation since 1995 (Puente et al., 1995). In contrast to our analysis, Puente et al. (1995) analyzed pooled milk samples without discussing individual variability. They quantified the sialic acid content of sheep milk at approximately 632 mg/kg on d 1 p.p., decreasing to approximately 209 mg/kg by d 30 p.p. Assuming an estimated density of sheep milk of 1 kg/L, these results align with the sialic acid quantification of this study when considering the sum of Neu5Ac and Neu5Gc between d 1 p.p. (512.9 ± 72.6 ng/µL) and d 30 p.p. (141.7 ± 6.2 ng/µL). Although the concentration of sialic acids and their decrease during lactation are consistent with our findings, the proportion of Neu5Gc was lower in the study by Puente et al. (1995): 63% on the first day, compared with 83% in mature milk. This discrepancy in results may result from the different breeds used because the 1995 study analyzed pooled milk samples from 6 Castellana-Awassi sheep, whereas this study analyzed the individual milk samples from 7 East Friesian sheep. In addition, the analytical technique used may influence the results. Puente et al. (1995) analyzed the sialic acid content by thin-layer chromatography, and we used an HPLC-based strategy. In summary, our results demonstrate that the amount of sialic acid in sheep milk during lactation follows the same trend as that observed in the milk of dairy cows and water buffaloes. However, the Neu5Gc:Neu5Ac ratio is reversed in these large ruminants, standing at approximately 10:90 instead of 90:10. This strongly suggests that Neu5Gc plays a unique role in sheep.

Furthermore, the change in polySia content in milk during lactation was analyzed. Polysialic acid is known to interact with and modulate the activity of numerous bioactive molecules. A notable example in milk is lactoferrin. In recent years, interest in immune-boosting dietary supplements, and specifically in animal colostrum and milk components such as lactoferrin, has increased rapidly (Kowalczyk et al., 2022). Lactoferrin is a multipotent glycoprotein found in most bodily fluids and tissues that exhibits antiviral, anti-inflammatory, and anticancer properties (Vogel, 2012; Zlatina and Galuska, 2021). In farm animals, sheep have the highest concentration of lactoferrin in milk, at 0.7 to 0.9 g/kg, which is closest to the concentration found in human milk, at 0.9 to 1.7 g/kg (Alichanidis et al., 2016). Previous work from our group demonstrated that linear homopolymers of Neu5Ac (polySia) can bind to lactoferrin, positively modulating its anti-inflammatory capacity without inactivating its antimicrobial activity (Kühnle et al., 2019a,b,c). However, the polysialylation status of milk from farm animals is largely unknown. Our group previously only detected polySia at levels close to the detection limit in mature sheep milk (Hinterseher et al., 2022), this study is the first to analyze the polySia content during lactation.

To this end, the Neu5Ac polymers in sheep colostrum and milk were first isolated by affinity purification using the polySia binding site of inactivated endoneuraminidase (**endoN**), which was coupled to tosyl-activated Dynabeads M-280 (Life Technologies, Oslo, Norway), as previously described (Kühnle et al., 2019c). The isolated polySia was then dried in a vacuum concentrator and stored at −20°C until further analysis.

Western blot analysis was performed to monitor polySia during lactation. The isolated polySia samples were therefore dissolved in radioimmunoprecipitation assay (RIPA) buffer (Cell Signaling Technology, Leiden, the Netherlands). An aliquot of each sample was treated with active endoN as a negative control, followed by incubation at 37°C for 1 h to degrade polySia. All samples were mixed with 5× Pierce Lane Marker Reducing Sample Buffer (Thermo Fisher Scientific, Waltham, MA) and heated at 60°C for 5 min. The samples were then separated by SDS-PAGE (7% gel) under reducing conditions and subsequently transferred onto a polyvinylidene fluoride (PVDF) membrane. Polysialic acid was identified using mAb 735 as the primary antibody, which binds specifically to the homopolymers of Neu5Ac in polySia. To visualize the binding of mAb 735, a secondary antibody of horseradish peroxidase-conjugated donkey anti-mouse IgG (Jackson ImmunoResearch Laboratories, Inc., West Grove, PA) was used. The chemiluminescence signal was detected using ECL Prime (Cytiva, Little Chalfont, UK) according to the manufacturer's instructions, and was then recorded using the ChemDoc MP Imaging System (Bio-Rad, Feldkirchen, Germany).

The western blot analysis revealed a strong signal for polySia in sheep colostrum and a rapid decrease of polySia during the first days of lactation, with no visible signal in mature milk (Figure 2A). This is consistent with the decrease in the Neu5Ac concentration during lactation of sheep, as well as with polySia reports for dairy cows (Hinterseher et al., 2022).

**Figure 2:**
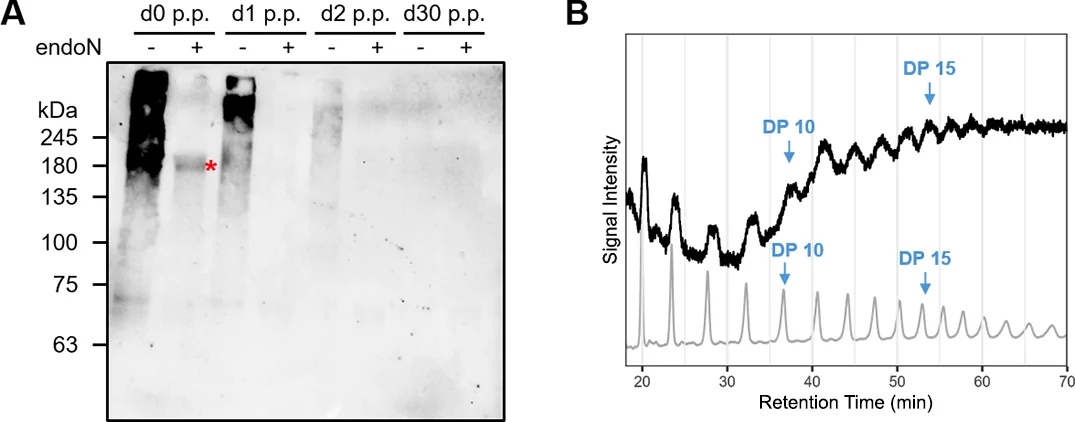
Analysis of polySia in sheep milk during early lactation. (A) Polysialylated proteins from sheep milk at d 0, 1, 2 and 30 p.p. were analyzed by western blotting using a monoclonal antibody against polySia. For the negative control, an aliquot of each sample was incubated with endoneuraminidase (endoN) to degrade polySia. The asterisk indicates an unspecific band. (B) The DP of polySia chains from sheep colostrum at d 0 p.p. (black line chromatogram) was analyzed using anion-exchange chromatography. Colominic acid was used to determine the chain lengths (gray line chromatogram). The signal intensity of the colominic acid peaks was reduced for the combined display of both chromatograms. The DP of selected peaks is indicated.

Because the interaction and activity of polySia depend on its chain length, the degree of polymerization (**DP**) was analyzed using an anion-exchange HPLC strategy (Kühnle et al., 2019b). For chain length analysis, polySia chains must be released from glycoconjugates under acidic conditions. They are then fluorescently labeled with DMB to achieve the required sensitivity (Inoue et al., 2001). Although these conditions primarily cleave the polySia-glycoconjugate linkage, partial internal hydrolysis of the sialic acid polymer is unavoidable because of the acidic conditions, which are necessary for both release and labeling (Inoue et al., 2001; Inoue and Inoue, 2003). Thus, the resulting chromatograms display signals from fragmented and intact chains. Nevertheless, the results are sufficient to determine whether a critical chain length exists in the analyzed samples that ensures the biological activity of polySia.

For HPLC analysis, the dried samples were dissolved in 40 µL of a DMB reagent solution. The same samples were used for this analysis as for western blotting. The samples were then derivatized by shaking overnight at 11°C. The reaction was stopped and lactonization was reversed by adding 10 µL of 1 *M* NaOH, followed by incubation at room temperature for 1 h. The samples were analyzed using a Nexera HPLC system (Shimadzu, Duisburg, Germany) with a DNAPac PA-100 column (4 × 250 mm; 13 µm; Thermo Fisher Scientific, Waltham, MA) as described previously (Hinterseher et al., 2022; Decloquement et al., 2023). As a reference standard, 0.05 µg of polySia from *Escherichia coli*, known as colominic acid (Gerbu, Heidelberg, Germany), was labeled and measured under the same conditions. Its chemical structure is identical to that of mammalian polySia (Galuska et al., 2007; Galuska et al., 2010).

Using the HPLC approach, sialic acid polymers consisting of more than 17 Neu5Ac residues were detected (see Figure 2B). Thus, the critical chain length necessary for interaction with various bioactive molecules, including lactoferrin and the antimicrobial peptide lactoferricin, is present in sheep milk (Kühnle et al., 2019a). This is the first time that the DP of polySia has been determined in sheep milk or colostrum. Yabe et al. (2003) first described polySia in milk using human colostrum, observing chains with up to 18 Neu5Ac residues. In contrast, we found a DP > 30 in human milk (Kühnle et al., 2019c). This was achieved by applying the same analysis strategy that we used here for the analysis of polySia in sheep milk. Thus, compared with human milk, sheep milk seems to contain shorter chains. As indicated by the relatively low signals, the polySia content was very low in sheep. One possible explanation for the low levels of polySia, and the low DP, is that human mature milk contains 900 ng/µL of Neu5Ac (Ret et al., 2025), which is more than 3 times the amount found in sheep colostrum. Because mammalian polysialyltransferases, the enzymes responsible for polySia synthesis, can only use nucleotide activated Neu5Ac and not Neu5Gc as a substrate (Decloquement et al., 2023), the synthesis of polySia might be limited. The fact that polySia was synthesized despite the low levels of Neu5Ac and that this synthesis requires considerable energy, suggests that it also plays an important role in the development of sheep offspring, particularly during the first few days of life. However, aside from its suggested role in modulating lactoferrin activity and serving as a reservoir for antimicrobial peptides, little is known about its other potential functions in milk.

In summary, the results demonstrate that the sialic acids Neu5Gc and Neu5Ac, as well as polySia, decreased rapidly during the early lactation phase in sheep, as has been observed in other ruminants. However, in contrast to large ruminants, Neu5Gc was the most abundant sialic acid at all analyzed time points. Thus, a distribution of sialic acids specific to sheep takes place in milk during lactation, and this has not yet been found in any other mammals.

It should be noted that for each conversion of Neu5Ac to Neu5Gc a NAD(P)H molecule is required (Schauer, 2004). The formation of high levels of Neu5Gc in sheep is therefore associated with high energy consumption. Such energy-intensive processes only become established during evolution if they confer an advantage to the animal. Thus, the high levels of Neu5Gc found in sheep colostrum and milk suggest that it plays an essential role in the development of their offspring. There are numerous possible reasons why Neu5Gc has taken on this dominant role over Neu5Ac in sheep. One possibility is the blocking of binding receptors of pathogens. Sialylated glycans in milk, whether bound or free, such as milk oligosaccharides, play a key role in preventing glycan-binding pathogens from attaching to the glycocalyx of the epithelium (Gagneux et al., 2022). It is likely that pathogens specific to sheep primarily possess Neu5Gc-binding receptors, which can be inhibited by glycans containing Neu5Gc in milk, thereby preventing invasion. Furthermore, sialic acids serve as a substrate for numerous bacteria (Hashimoto et al., 2025). It is also possible that Neu5Gc has beneficial effects in sheep by promoting a healthy microbiome. However, there is currently no research data available on either of the 2 possible roles of Neu5Gc. Elucidating the biological function of Neu5Gc in sheep could provide new insights into optimizing the rearing of their offspring.

From a human health perspective, it is important to note that the colostrum and transitional milk of sheep contains high levels of Neu5Gc, which may trigger proinflammatory responses. Because colostrum is consumed as a dietary supplement, attention should be paid to its high proinflammatory Neu5Gc content.

## References

[bib1] Albrecht S., Lane J.A., Mariño K., Al Busadah K.A., Carrington S.D., Hickey R.M., Rudd P.M. (2014). A comparative study of free oligosaccharides in the milk of domestic animals. Br. J. Nutr..

[bib2] Alichanidis E., Moatsou G., Polychroniadou A., Tsakalidou E., Papadimitriou K. (2016). Non-Bovine Milk and Milk Products.

[bib3] Bode L. (2015). The functional biology of human milk oligosaccharides. Early Hum. Dev..

[bib4] Chen Q., Mueed A., Zhu L., Deng Z., Peng H., Li H., Zhang B. (2024). HPLC-QQQ-MS/MS-based authentication and determination of free and bound sialic acids content in human, bovine, sheep, goat milk, and infant formula. J. Food Sci..

[bib5] Colley K.J., Kitajima K., Sato C. (2014). Polysialic acid: Biosynthesis, novel functions and applications. Crit. Rev. Biochem. Mol. Biol..

[bib6] Decloquement M., Venuto M.T., Cogez V., Steinmetz A., Schulz C., Lion C., Noel M., Rigolot V., Teppa R.E., Biot C., Rebl A., Galuska S.P., Harduin-Lepers A. (2023). Salmonid polysialyltransferases to generate a variety of sialic acid polymers. Sci. Rep..

[bib7] Dhar C., Sasmal A., Varki A. (2019). From “serum sickness” to “xenosialitis”: Past, present, and future significance of the non-human sialic acid Neu5Gc. Front. Immunol..

[bib8] Gagneux P., Hennet T., Varki A., Gagneux P., Hennet T., Varki A. (2022). Essentials of Glycobiology.

[bib9] Galuska S.P., Geyer H., Bleckmann C., Rohrich R.C., Maass K., Bergfeld A.K., Muhlenhoff M., Geyer R. (2010). Mass spectrometric fragmentation analysis of oligosialic and polysialic acids. Anal. Chem..

[bib10] Galuska S.P., Geyer R., Mühlenhoff M., Geyer H. (2007). Characterization of oligo- and polysialic acids by MALDI-TOF-MS. Anal. Chem..

[bib11] Hara S., Takemori Y., Yamaguchi M., Nakamura M., Ohkura Y. (1987). Fluorometric high-performance liquid chromatography of N-acetyl- and N-glycolylneuraminic acids and its application to their microdetermination in human and animal sera, glycoproteins, and glycolipids. Anal. Biochem..

[bib12] Hashimoto R., Nishiyama K., Namai F., Suzuki K., Sakuma T., Fukuda I., Sugiyama Y., Okano K., Shanoh T., Toyoshi E., Ohgi R., Saha S., Tsuchida S., Nishiyama E., Mukai T., Furukawa M., Nochi T., Villena J., Ikeda-Ohtsubo W., Yoshioka G., Nakazaki E., Suda Y., Kitazawa H. (2025). Milk sialyl-oligosaccharides mediate the early colonization of gut commensal microbes in piglets. Microbiome.

[bib13] Hinterseher J., Günther J., Zlatina K., Isernhagen L., Viergutz T., Wirthgen E., Hoeflich A., Vernunft A., Galuska S.P. (2022). Milk polysialic acid levels rapidly decrease in line with the N-acetylneuraminic acid concentrations during early lactation in dairy cows. Biology (Basel).

[bib14] Hobbs M., Jahan M., Ghorashi S.A., Wang B. (2021). Current perspective of sialylated milk oligosaccharides in mammalian milk: Implications for brain and gut health of newborns. Foods.

[bib15] Inoue S., Inoue Y. (2003). Ultrasensitive analysis of sialic acids and oligo/polysialic acids by fluorometric high-performance liquid chromatography. Methods Enzymol..

[bib16] Inoue S., Lin S.L., Lee Y.C., Inoue Y. (2001). An ultrasensitive chemical method for polysialic acid analysis. Glycobiology.

[bib17] Isernhagen L., Galuska C.E., Hoeflich A., Galuska S.P. (2025). Characterization of the milk oligosaccharide spectrum in water buffaloes during early lactation. Carbohydr. Polym..

[bib18] Isernhagen L., Galuska C.E., Vernunft A., Galuska S.P. (2024). Structural characterization and abundance of sialylated milk oligosaccharides in Holstein cows during early lactation. Foods.

[bib19] Kowalczyk P., Kaczyńska K., Kleczkowska P., Bukowska-Ośko I., Kramkowski K., Sulejczak D. (2022). The lactoferrin phenomenon—A miracle molecule. Molecules.

[bib20] Kühnle A., Galuska C.E., Zlatina K., Galuska S.P. (2019). The bovine antimicrobial peptide lactoferricin interacts with polysialic acid without loss of its antimicrobial activity against *Escherichia coli*. Animals (Basel).

[bib21] Kühnle A., Lütteke T., Bornhöfft K.F., Galuska S.P. (2019). Polysialic acid modulates the binding of external lactoferrin in neutrophil extracellular traps. Biology (Basel).

[bib22] Kühnle A., Veelken R., Galuska C.E., Saftenberger M., Verleih M., Schuppe H.-C., Rudloff S., Kunz C., Galuska S.P. (2019). Polysialic acid interacts with lactoferrin and supports its activity to inhibit the release of neutrophil extracellular traps. Carbohydr. Polym..

[bib23] Puente R., Garcia-Pardo L.A., Rueda R., Gil A., Hueso P. (1995). Ewes' milk: Changes in the contents of gangliosides and sialic acid during lactation. J. Dairy Res..

[bib24] Ret D., Gentile S.A., Rohrhofer J., Koidl L., Cozzi F., Untersmayr E. (2025). Species-specific N-glycan patterns in animal and human breast milk samples. Front. Nutr..

[bib25] Robinson R.C. (2019). Structures and metabolic properties of bovine milk oligosaccharides and their potential in the development of novel therapeutics. Front. Nutr..

[bib26] Schauer R. (2004). Sialic acids: Fascinating sugars in higher animals and man. Zoology (Jena).

[bib27] Schnaar R.L., Gerardy-Schahn R., Hildebrandt H. (2014). Sialic acids in the brain: Gangliosides and polysialic acid in nervous system development, stability, disease, and regeneration. Physiol. Rev..

[bib28] Tangvoranuntakul P., Gagneux P., Diaz S., Bardor M., Varki N., Varki A., Muchmore E. (2003). Human uptake and incorporation of an immunogenic nonhuman dietary sialic acid. Proc. Natl. Acad. Sci. U S A.

[bib29] Varki A. (2010). Uniquely human evolution of sialic acid genetics and biology. Proc. Natl. Acad. Sci. U S A.

[bib30] Vogel H.J. (2012). Lactoferrin, a bird's eye view. Biochem. Cell Biol..

[bib31] Yabe U., Sato C., Matsuda T., Kitajima K. (2003). Polysialic acid in human milk: CD36 is a new member of mammalian polysialic acid-containing glycoprotein. J. Biol. Chem..

[bib32] Zlatina K., Galuska S.P. (2021). The N-glycans of lactoferrin: More than just a sweet decoration. Biochem. Cell Biol..

